# Surgical Techniques for Non-Small-Cell Lung Cancer After Neoadjuvant Chemo-Immunotherapy: State of Art and Review of the Literature

**DOI:** 10.3390/cancers17040638

**Published:** 2025-02-14

**Authors:** Beatrice Trabalza Marinucci, Massimiliano Mancini, Alessandra Siciliani, Fabiana Messa, Giorgia Piccioni, Antonio D’Andrilli, Giulio Maurizi, Anna Maria Ciccone, Cecilia Menna, Camilla Vanni, Matteo Tiracorrendo, Erino Angelo Rendina, Mohsen Ibrahim

**Affiliations:** 1 Department of Thoracic Surgery, Sant’Andrea Hospital, Sapienza University, 00189 Rome, Italy; alessandrasiciliani@gmail.com (A.S.); fabiana.messa@uniroma1.it (F.M.); giorgia.piccioni@uniroma1.it (G.P.); adandrilli@hotmail.com (A.D.); giulio.maurizi@uniroma1.it (G.M.); cicconeam@gmail.com (A.M.C.); cmenna@ospedalesantandrea.it (C.M.); camillavanni.1@gmail.com (C.V.); matteo.tiracorrendo@ospedalesantandrea.it (M.T.); erinoangelo.rendina@uniroma1.it (E.A.R.); mohsen.ibrahim@uniroma1.it (M.I.); 2 Department of Histopathology, Sant’Andrea Hospital, Sapienza University, 00189 Rome, Italy; mamancini@ospedalesantandrea.it

**Keywords:** non-small-cell lung cancer, lung surgery, neoadjuvant immunotherapy, resectability

## Abstract

Non-small-cell lung cancer (NSCLC) accounts for 80–85% of all lung cancers. Approximately 20% of patients with NSCLC are diagnosed with stage IIIA–IIIB disease, for which the optimal treatment remains unclear. Meta-analyses reveal that neoadjuvant/perioperative ICI–chemotherapy significantly improves pathological complete response (pCR), overall survival (OS), major pathological response (MPR), and R0 rate compared to standard neoadjuvant chemotherapy. Resectability is achieved when R0 resection can be performed after surgery. Radiographic downstaging often does not correspond to surgical downstaging. In fact, intra-operative fibrosis due to chemo-immunotherapy (synonymous with ICI–chemotherapy) can create adhesions and consequent difficult planes for dissection. Thus, pneumonectomy cannot be avoided. Even the suspicion of N2 after neoadjuvant treatment is considered a limitation of upfront surgery because of the risk of pneumonectomy. The aim of this review is to explore the literature on the technical strategies for surgical excision of NSCLC after chemo-immunotherapy, addressing even the most challenging scenarios.

## Simple Summary

Recent trials have demonstrated that the addition of immune checkpoint inhibitors (ICIs) to chemotherapy in the neoadjuvant setting improves response and survival outcomes in intention-to-treat (ITT) populations. Several trials describe the operative difficulties related to the treatment in terms of intra-operative findings (fibrosis and adhesions), rate of conversion, and post-operative complications. This review aims to summarize the main points related to surgery after neoadjuvant chemo-immunotherapy, highlighting strategies to maximize the operability of tumors while minimizing the need for invasive and potentially debilitating surgeries such as pneumonectomy, as well as providing a perspective to overcome the surgical issue of lack of resectability after neoadjuvant ICI–chemotherapy.

## 1. Introduction

Lung cancer is the leading cause of cancer-related mortality worldwide and significantly contributes to reduced life expectancy.

Non-small-cell lung cancer (NSCLC) accounts for 80 to 85% of all lung cancers. Approximately 20% of patients with NSCLC are diagnosed with stage IIIA or IIIB disease. Treatment options for NSCLC include surgery, radiotherapy, chemotherapy, targeted therapy, and immunotherapy. Surgery remains the cornerstone of curative treatment for early and locally advanced NSCLC, with 5-year overall survival rates ranging from 92% for stage IA to 26% for stage IIIB [1].

Nevertheless, the optimal treatment for patients with stage IIIA or IIIB disease remains unclear, as stage III NSCLC is a condition characterized by different combinations of tumor size and nodal stations’ involvement [2].

Several recent trials have demonstrated that immune checkpoint inhibitors (ICIs) added to chemotherapy in the neoadjuvant setting may improve pathological response, expanding the population who can benefit from neoadjuvant therapy before surgery. To date, neoadjuvant therapy based on ICI–chemotherapy is being increasingly indicated as standard of care for resectable NSCLC in NCCN, ESMO, and ASCO guidelines [3].

Recent advancements in managing resectable NSCLC have markedly improved with perioperative approvals, marking a new era in which molecularly targeted therapies and immune checkpoint inhibitors enhance treatment efficacy.

Meta-analyses reveal that neoadjuvant/perioperative chemo-immunotherapy (ICI–chemotherapy) significantly improves pathological complete response (pCR), overall survival (OS), major pathological response (MPR), and R0 resection rate [4].

Resectability is defined as the achievement of free negative margins for malignancy, defined as R0 resection, after complete tumor excision. Complete resection (R0) even includes systematic node dissection. However, the real resectability after pathological response radiologically evaluated is still difficult to assess [5].

For patients who are borderline resectable because of the anatomical location of the tumor, it is unclear whether radiographic downstaging actually leads to surgical downstaging. Intra-operatively, neoadjuvant (NA) therapy-induced fibrosis generates complex dissection plans, making it difficult to distinguish fibrosis from residual malignancy, and a pneumonectomy could often not be avoided.

To date, even the suspicion of N2 after neoadjuvant treatment is considered a limitation of upfront surgery because of the risk of pneumonectomy.

The aim of this review is to explore the literature on the technical strategies for surgical excision of NSCLC after ICI–chemotherapy, addressing even the most challenging scenarios. In addition, unsolved challenges such the importance of preserving lung function, highlighting strategies to maximize the operability of tumors while minimizing the need for invasive and potentially debilitating surgeries such as pneumonectomy, will be argued, and we will also provide our perspective to overcome surgical issues of unresectability after neoadjuvant ICI–chemotherapy.

## 2. Materials and Methods

A narrative literature review was conducted using PubMed, Medline, and Google Scholar for English-language articles published from 2019 to 2024. The search terms included *neoadjuvant chemo-immunotherapy*, *surgical complexity after chemo-immunotherapy*, *resectable NSCLC*, and *unresectable NSCLC*.

**Study Selection Criteria:**
  - Inclusion criteria: Original articles, reviews, and clinical trials describing surgical techniques, oncological resections, and post-operative complications after neoadjuvant ICI–chemotherapy.
  - Exclusion criteria: Editorials, commentaries, case reports, and articles without available full texts [6].

**Selection Process:**
  - Initial screening based on titles (50 studies selected).
  - Abstract screening (duplicates removed).
  - Full-text review (final selection: 30 studies).
  - Data extraction and synthesis.

**Figure 1:**
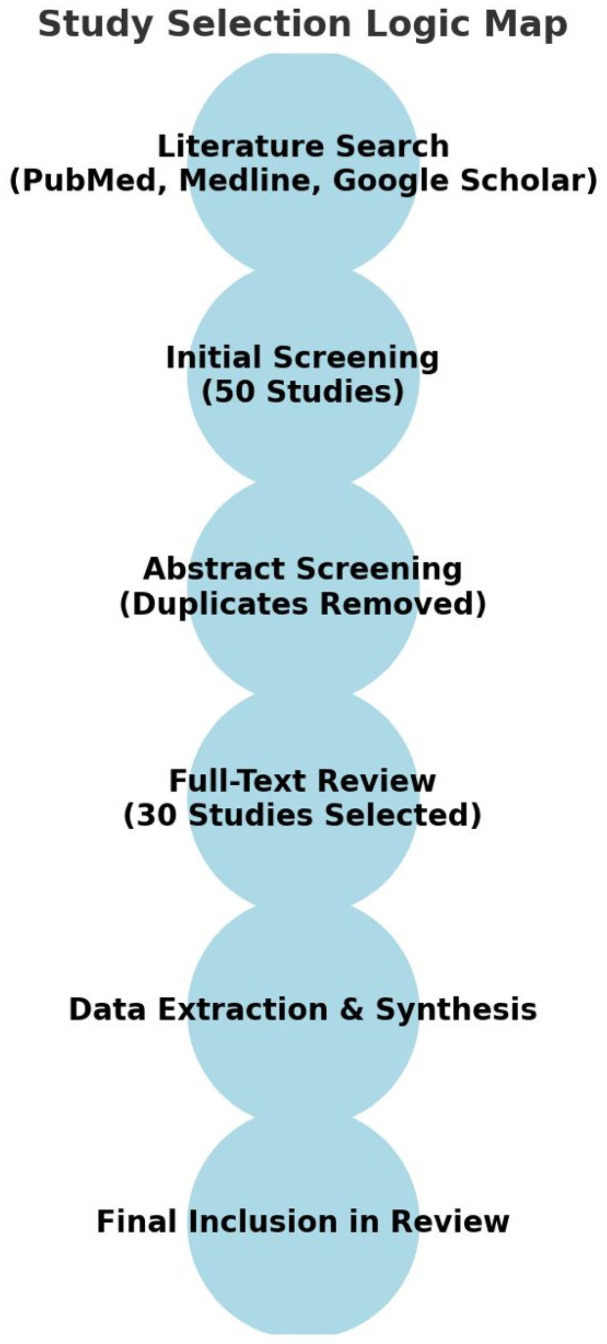
Study selection logic map.

Initially, 50 studies were included. After screening abstracts and full texts for relevance, 30 studies were finally selected for review (Figure 1).

## 3. Results

1. Clinical Endpoints

The complexity of surgery related to ICI–chemotherapy NA treatment for resectable NSCLC has been widely described. To date, clinical endpoints describing the impact of neoadjuvant ICIs on surgery are not well defined. It is necessary to point out specific endpoints related to surgical difficulty to homogenize the findings of clinical trials, thus evaluating the real impact of the treatment on surgery, and to predict in advance any technical difficulties.

Proposed intra-operative endpoints are now considered: blood loss, duration of surgery, and type of surgery.

Lee et al. [7] proposed a scoring system to define surgical complexity based on lymphadenopathy and fibrosis. The aim of the score was to assess an objective quantitation of technical surgical difficulty after neoadjuvant chemo-immunotherapy. They introduced a four-point complexity score system scale based on size of lymphadenopathy (<1 cm, 1–2 cm, 2–3 cm, or >3 cm), peripheral fibrosis (mild fibrosis, moderate fibrosis increasing surgery complexity, severe fibrosis requiring conversion, or severe fibrosis resulting in unresectability), site of lung cancer (central or peripheral), and perihilar/mediastinal adhesions (mild fibrosis, moderate fibrosis increasing surgery complexity, severe fibrosis requiring conversion, or severe fibrosis resulting in unresectability). A score of 2 is assumed to be a “normal dissection”.

Sepesi et al. [8] in a NEOSTAR study included other variables to define intra-operative technical difficulty, namely time of surgery from the cut to closure, intra-operative blood loss, and conversion rates for the minimally invasive approaches.

Zhang et al. described intra-operative surgical difficulty based on a score system that considers level of intrathoracic adhesion (0 = no adhesion; 1 = mild/moderate adhesions; 2 = severe adhesions), difficulty of nodes’ dissection (0 = normal; 1 = hard; 2 = extremely hard), grade of tumor invasion (0 = no peripheral invasion; 1 = mild invasion of pulmonary artery (PA) and/or bronchus; 2 = severe invasion of the main PA and/or bronchus), and difficulty of the whole procedure (0 = normal; 1= hard; 2 = extremely hard). Each variable consists of three degrees according to the score, and total difficulty was defined according to a final evaluation [9].

Clinical endpoints predicting the response to therapy are matter of interest for the prognosis and the overall survival after surgery. In fact, ongoing trials are looking for potential biomarkers to facilitate patient selection and risk stratification. Zaric et al. [10] reported a potential connection between survival and PD-1 tissue expression; however, PD-L1 to date cannot be considered an independent prognostic factor for cancer recurrence. Also, the tumor mutation burden (TMB) was identified as a potential predictive biomarker after neoadjuvant chemo-immunotherapy [11]. ctDNA can be considered a promising marker to screen pathological response, thanks to the advances in liquid biopsy technologies; as reported by McDonald et al. [12], pre-operative ctDNA was found to be a strong predictor of relapse-free survival and overall survival after complete resection of pulmonary tumors. Finally, studies on the microbiome before and after neoadjuvant immunotherapy are interesting: the gut microbiota, galectin-3, and the intensity of CD8 cell infiltration are proposed as predictive biomarkers of survival [13].

Defining surgical complexity remains a challenge. Several studies propose scoring systems for intra-operative difficulty:

- Lee et al. (2021) developed a score based on lymphadenopathy and fibrosis severity.
- Sepesi et al. (NEOSTAR study, 2019) introduced a four-point scale considering tumor location, fibrosis, and adhesion severity.
- Zhang et al. (2021) classified surgical difficulty based on intrathoracic adhesion levels, nodal dissection difficulty, and vascular invasion.

A comparative table summarizing these scoring systems has been added (Table 1).

2. Unresectability after chemo-immunotherapy

Incomplete resection negatively affects overall survival (OS) irrespective of tumor stage. Unresectability related to the treatment could be due to progression of the disease or highly severe pleural and/or mediastinal fibrosis. In recent trials, the rates of intra-operative unresectability after neoadjuvant ICI therapy (range from 0% to 5%) and after ICI plus chemotherapy (range from 0% to 10%) were comparable. Kwiatkowski et al. [14] and then Lee et al. [15] in the LCMC3 trial described 5% unresectability in a total of 159 patients that underwent surgery for NSCLC stage IB–IIIB after 30–50 days of atezolizumab in a neoadjuvant setting. In this study, 5% of patients were found to have pre-operative progression of the disease in stage IIIA, while 6% were found to have intra-operative progression in stage IIIA and/or IIIB. In CheckMate 159, one patient (5%) with stage IIIA disease was found to have tracheal invasion after neoadjuvant treatment with nivolumab [3].

Unresectability post-ICI–chemotherapy is influenced by progressive disease, pleural/mediastinal fibrosis, and vascular adhesions. Studies report intra-operative unresectability rates of 5–10%:

- The LCMC3 trial (Kwiatkowski et al., 2019) reported a 5% unresectability rate post-ICI-therapy.
- The CheckMate 159 trial (2019) found 5% of stage IIIA cases had intra-operative tracheal invasion.

These findings are presented in Table 2.

3. Oncological resections

Surgical resection after neoadjuvant chemo-immunotherapy could be performed both by an open approach (thoracotomy, sternotomy, clamshell, or hemiclamshell incision) and minimally invasive surgery (video- or robot-assisted thoracoscopic surgery).

The major concerns with the video-assisted thoracic surgery (VATS) approach after neoadjuvant immunotherapy are related to the risk of perihilar inflammation, overthrow of the planes, vascular fragility, intrathoracic adhesions, and the need for extended nodal dissection. Resection can be initially performed under VATS, and conversion to hybrid VATS or open surgery should be performed when necessary. Conversion is justified to obtain an R0 resection and to achieve the best safety of the patient if intra-operative complications arise.

In fact, thoracotomy does not appear to significantly affect morbidity and early mortality rates [23].

Sepesi et al. [8] described a cohort of 37 patients who underwent surgery after ICI, of which 73% underwent lung resection by thoracotomy, 19% by VATS, and 8% by robotic-assisted thoracoscopic surgery (RATS).

In a retrospective study, Zeng et al. [16] described a comparative analysis between the use of VATS and RATS in a total of 220 patients undergoing neoadjuvant chemo-immunotherapy from May 2020 to August 2022: 78 patients were operated on using the VATS approach and 142 by RATS. RATS was performed with the Da Vinci Xi surgery system (Intuitive Surgical, Inc., Mountain View, CA, USA), using three arms. The 28.2% of patients who underwent VATS surgery and the 7.5% of patients who underwent RATS needed conversion. The main causes of conversion were dense adhesion, subsequent fibrosis, and intra-operative bleeding. The time of surgery for VATS was shorter compared to RATS (176.94 ± 74.974 min vs. 197.28 ± 70.945 min, *p* = 0.048). Complications were detected in 26 patients operated on by VATS and 45 patients operated on by RATS, with pneumonia being the most common in both groups. The Da Vinci RATS employed more sophisticated equipment compared to traditional VATS, facilitating lymph node assessment [16].

Anatomical resections (such as segmentectomy, lobectomy, and bilobectomy) are strongly preferred, while pneumonectomy is preferentially avoided due to having the poorest prognosis. Lobectomy is the most common resection performed in several phase 2 studies (lobectomy was described in 65–93% of the cases of monotherapy or dual immunotherapy and in 73–93% of cases after chemo-immunotherapy, respectively) [Table 2].

However, pneumonectomy was still required in 9% to 17% of patients because a significant downstaging after neoadjuvant therapy is not always observed; in recent phase-3 clinical trials, this figure was 17% in CheckMate 816, 11% in Keynote-671, and 9% in Neotorch and Rationale-315 [24]. A limitation of the trials is that, unfortunately, none included a specific intra-operative evaluation of the technical difficulties caused by neoadjuvant treatment, so it is difficult to assess which is the final indication for pneumonectomy. Moreover, previous reports from clinical trials showed a mortality rate around 7% for patients who underwent pneumonectomy after neoadjuvant chemo-immunotherapy.

Some studies demonstrated that even extended resections are feasible in patients who underwent neoadjuvant immune or chemo-immunotherapy: Romero et al. described that in their cohort, pneumonectomy could be avoided in six cases (14%) using extended resections [25].

Liang et al. described a retrospective analysis of 108 patients who underwent sleeve lobectomy (with added angioplasty or intrapericardial resections when necessary) between December 2016 and December 2019; among them, 10 patients received neoadjuvant chemotherapy (11%), and 10 patients were treated with neoadjuvant ICI–chemotherapy (nivolumab, pembrolizumab, or sintilimab). No increased incidence of post-operative complications was found in the ICI–chemotherapy group [17].

Another series by Zhu et al. described sleeve resection in 23 patients, performed after 4–6 weeks from the end of the neoadjuvant therapy. Surgical access between VATS and thoracotomy was chosen according to the preference of the surgeon. When the lesions involved even the main vascular trunks, double-sleeve resection (bronchus and vessel) was performed. All patients operated on achieved a free margin resection (R0 resection). Post-operative complications were observed in three patients, and no statistically significant difference was observed between VATS and thoracotomy. Sleeve resection after neoadjuvant chemo-immunotherapy showed comparative outcomes in terms of survival compared to cases where intervention was required for patients who did not undergo neoadjuvant chemo-immunotherapy [18]. Post-operative complications were described in three (13%) patients in the thoracotomy group, without difference among patients who underwent neoadjuvant chemo-immunotherapy or not; thus, no more anastomosis-related complications related to the neoadjuvant treatment were observed in this study [26].

Chen et al. in 2020 [19] reported the feasibility of sleeve lobectomy after neoadjuvant chemo-immunotherapy, showing no difference in complication rate compared to surgery alone. Neoadjuvant immunotherapy was not associated with an increase in post-operative pulmonary complications. There were no major anastomotic or other complications in the ICI cohort, but there were four anastomotic complications in the chemotherapy cohort.

Preferred surgical strategies include lobectomy, sleeve lobectomy, and extended resections to avoid pneumonectomy. Studies evaluating minimally invasive techniques found the following:

- VATS conversion rates: 28.2% (Zeng et al., 2023) vs. 11.4% (CheckMate 816).
- Robotic-assisted (RATS) conversion rates: 7.5% (Zeng et al., 2023).
- Thoracotomy remains necessary in 59–73% of cases.
- CheckMate 816 (2022) and Neotorch (2023) showed that pneumonectomy was still required, respectively, in 17% and 9% of cases.

Tables summarizing conversion rates, surgical approaches, and post-operative complications have been expanded.

4. Node dissection

Several retrospective studies demonstrated a significant association between the number of lymph nodes harvested and the overall survival, especially considering the clinical N1–2 stage. The main difficulty in the neoadjuvant ICI–chemotherapy is the technical difficulty in lymph node harvesting because of the fibrotic effects related to the treatment.

Systematic node dissection must always be performed, including both hilar and mediastinal nodes.

Right-sided lesions require a dissection involving at least levels 4R, 7, 10R, and 11R, while left-sided lesions require at least harvesting levels of 5/6, 7, 10L, and 11L.

For lower lobe cancers, lymph node dissection should involve levels 8 and 9.

A study by Zhang et al. [9] demonstrated a significant difference in the number of lymph nodes harvested between the group of patients who underwent neoadjuvant therapy and the non-neoadjuvant group because of the intra-operative harvesting difficulty; in fact, severe adhesion or vascular invasion by the lymph nodes can limit the systematic dissection or may cause the nodes dissected to be smaller and/or fragmented.

Liang et al. found a smaller number of lymph nodes harvested in the chemo-immunotherapy group compared to the chemotherapy group: 51% at the N2 level. Comparable to Zheng’s study, they too attributed the smallest lymph node number harvested to the intra-operative difficulty to perform the systematic dissection. Another problem in lymph node dissection is that some medicaments used in ICI–chemotherapy, such as sintilimab, could cause tissue changes such as fusion or shrinking [16].

The SAKK 16/14 trial is the largest published trial of perioperative anti-PD-L1 therapy in addition to neoadjuvant chemotherapy in patients with resectable stage IIIA(N2) NSCLC; in this study, the addition of perioperative durvalumab to neoadjuvant chemotherapy with cisplatin and docetaxel resulted in a high 1-year event-free survival (EFS) rate of 73%, and mediastinal lymph node dissection was confirmed as one of the most important prognostic factors [22].

The importance of systematic lymph node dissection (SND) after ICI–chemotherapy has been confirmed in several studies as a prognostic factor, highlighting the principal station required for:

- Right-sided lesions: including levels 4R, 7, 10R, and 11R.
- Left-sided lesions: including levels 5/6, 7, 10L, and 11L.
- Lower lobe cancers: including levels 8 and 9.

Challenges for extensive node dissection include adhesions and fibrosis, leading subsequently to fewer harvested nodes after NA ICI–chemotherapy (Liang et al., 2021).

5. Conversion rate

The correct approach should be chosen considering the characteristics of the tumor, patients’ comorbidities, and surgeon self-confidence. Variables related to surgical difficulty include extensiveness of resection, type of anatomical resection, and rate of conversion from a minimally invasive surgery.

The minimally invasive approach appears to be feasible in patients who underwent surgery after chemo-immunotherapy, but attention should be paid to patients with bulky tumors, suspicious infiltration of the mediastinum, and/or pulmonary vessels [27].

In CheckMate 159, conversions to thoracotomy were performed in the 50% of patients staged I and/or IIA, due to intrathoracic adhesions.

Bott et al. [23] described a phase I trial including 20 patients affected by NSCLC undergoing surgery after nivolumab in a neoadjuvant setting; the conversion rate was described in 7 of 13 minimally invasive procedures (VATS or RATS), in 25% of the patients with stage I, 50% of patients with stage IIA, and 71% with stage IIB or IIIA. The indication for conversion rate was principally hilar inflammation, dense adhesions related to the treatment surrounding hilum, fissure, or mediastinal nodal stations.

In the cohort by Sepesi et al. [8], 17% of the 12 patients who underwent a minimally invasive approach had conversion.

Pataer et al. [28] reported the efficacy of the minimally invasive approach without the need of conversion in 67% of patients.

Nevertheless, in the CheckMate 816, dealing with patients affected by stage IB–IIIA NSCLC, Forde et al. [3] described a minimally invasive surgery performed in 30% of the patients, with a conversion rate of 11% for the chemo/immunotherapy patients vs. 16% for chemotherapy alone; conversion was not associated to the stage of the tumor.

In the study by Bott et al. [29], conversion to thoracotomy was required in 20% of the five patients who underwent anatomic resections via the minimally invasive approach, one of them after robotic-assisted right upper lobectomy. 

In the NADIM trial, in which 46 patients with stage IIIA NSCLC were enrolled for treatment with neoadjuvant chemotherapy and nivolumab, 89.1% underwent surgery, and the conversion rate from VATS to open surgery was described in 19% of cases [30].

6. Surgery difficulties

Immunotherapy-related adverse events may delay surgery and/or increase the risk of intra-operative complications and finally may result in tumor progression.

Data from recent trials on neoadjuvant chemo-immunotherapy describe that the disease progression rate is around 10% in patients affected by higher NSCLC stages, such as stage IIIA.

Moreover, neoadjuvant chemo-immunotherapy may destroy tumor vascularization and the microenvironment, subsequently producing intrathoracic adhesions and/or fibrosis, thus increasing surgical difficulty and the total duration of surgery [31].

Figueroa et al. [24] described the results of some of the most recent clinical trials (CheckMate 816, Keynote-671, Aegean, and Rationale-315), considering the conversion rate and complications. Unfortunately, none of the most recent phase 3 trials on neoadjuvant chemo-immunotherapy describe in detail the technical intra-operative challenges caused by the neoadjuvant treatment itself. In fact, technical difficulties were principally caused by post-therapy inflammation, intrathoracic adhesions, fibrosis in the fissure and/or around the hilum, and mediastinal nodal stations.

Considering that neoadjuvant therapy can worsen a patient’s physical condition as well as performance status, Dickhoff et al. [32] proposed the following as principal factors of worsening condition: tissue inflammation after therapy, fibrosis consequent to therapy, and finally the hypervascularization of the tissues. However, to date, objective metrics to quantify the extent of intrathoracic adhesions and fibrotic changes related to the neoadjuvant treatment are still difficult to assess.

In fact, hilar fibrosis is a common finding, often affecting the hilum but also the pulmonary artery, vein, and trachea, increasing frailty. Fibrosis often affects hilar and mediastinal lymph nodes, increasing the surgeon’s perception of the complexity of the resection.

Among tissue changes caused by neoadjuvant chemo-immunotherapy, the most frequent changes reported are the thickening of the vessel wall and the thickening of the tissue around the tumor, thus affecting the vascularization of the surrounding parenchyma. Even the fibrosis around the lymph nodes can lead to an increase in surgical intra-operative risks [33].

Compared to standard chemotherapy, ICI–chemotherapy determines a greater reduction in the elasticity of vessel walls, together with vascular wall degeneration, fibrinoid necrosis, and pulmonary interstitial exudation [34].

A NEOSTAR study provided a scale of surgical complexity based on a four-point complexity score, assuming a value of 2 as “normal dissection”, that is to say a lobectomy for stage I NSCLC. In the study, 40% of 37 surgeries were judged to be more difficult than usual (with assessed score 3), judged on the basis of the median operative time (147 min; range: 71–315 min) and median blood loss (100 mL; range: 50–1000 mL) [8].

The different studies analyzed agree that the main intra-operative surgical difficulties are related to post-therapy inflammation, intrathoracic adhesions, and fibrosis in the fissure or around the hilum and mediastinal nodal stations. There is a lack of objective metrics to quantify the tissue changes due to the therapy in literature, and one of the most interesting works in this vein is the NEOSTAR, providing a scale of surgical complexity based on the easiness of dissection, blood loss, and operative time.

7. Post-operative complications

Delayed surgery related to possible adverse events due to neoadjuvant immunotherapy is associated with poorer outcomes. Moreover, physical decline and worsening of performance status induced by neoadjuvant therapy may prolong post-operative recovery for the patients.

Some studies reported the rate of 30-day post-operative mortality in patients who underwent surgery after neoadjuvant therapy, which ranged from 3% to 10%. The main described complications in the same studies were found to be pneumonia (3–10%), bronchopleural fistula (2–5%), and prolonged air leak (9%) (Table 3). In the Keynote trial, which explored the use of pembrolizumab for metastatic NSCLC, pneumonia was described in 4% of patients. Similarly, in the CheckMate 012 trial, which examined the combination of ipilimumab and nivolumab in a similar patient population, pneumonia was described in 10% of patients.

Even the side effects of therapy (such as adrenal insufficiency and hyperthyroidism) might increase the risks of intra- and/or post-operative complications [29].

The French Intergroupe Francophone de Cancérologie Thoracique (IFCT)-1601 Ionesco trial [36] was a multicenter phase II, single-arm study evaluating neoadjuvant durvalumab alone for NSCLC staged IB and IIIA, excluding N2 involvement. The study included 46 patients; five patients died on the 94th post-operative day (three patients after lobectomy and two patients after pneumonectomy). The main causes of death were unexpected arterial damage, post-operative respiratory distress, tracheal fistula, and cardiac complications. The authors concluded that a better selection of patients for neoadjuvant immunotherapy would be required, and a score describing surgical technical difficulties should be developed to better select patients who really would benefit from NA treatment. Indeed, the study seems limited by some bias, such as the wide variability of the range between the last infusion of darvulumab and surgical resection, the heterogeneity of patient selection (multicenter study with the involvement of 20 active centers, small sample), and inclusion of patients (evidence of no response and progression in most cases).

Several degrees of post-operative complications are described in the different trials, with a major incidence of pneumonia, followed by broncho-pleural fistula and prolonged air leakage. The main limitation is related to the heterogeneity of the studies because post-operative complications are related to different therapy side effects, delayed surgery due to an adverse event during the NA treatment, and the surgical expertise of the different centers. Thus, no homogeneous consensus on the effective rate of post-operative complications after NA ICI–chemotherapy could be achieved due to the different conditions proposed by the trials.

## 4. Discussion

While many studies in the existing literature discuss the oncological outcomes of surgery following immune checkpoint inhibitor (ICI) and chemotherapy regimens, fewer have focused on the technical challenges that surgeons face in these cases. Our review sheds light on these difficulties, emphasizing how fibrosis, adhesions, and vascular changes contribute to increased surgical complexity.

We also highlight that minimally invasive approaches, while often preferred, tend to have higher conversion rates after ICI therapy due to these anatomical changes. Despite the technical hurdles, systematic lymph node dissection remains a crucial step in these procedures, ensuring comprehensive staging and disease management. Additionally, we explore the role of sleeve lobectomy as a viable alternative to pneumonectomy, offering a lung-sparing option in appropriately selected patients.

The clarification of specific surgical aspects, intra- and peri-operative complications, and outcomes could help surgeons in the surgical management of this set of patients. Moreover, a better understanding of the surgical difficulties and the different potentiality of surgery after chemo-immunotherapy, could provide a novel insight into the way the trials are set. In fact, this could guide the enrollment of patients into neoadjuvant therapy trials based on the concerns about surgical technical aspects, surgical complexity, and post-operative complications.

To date, the kind of patients who would benefit most from adjuvant immunotherapy therapy after surgery is not clear, and ongoing studies are exploring the field. Moreover, patients considered to have unresectable disease—for example, those with T4 tumors, direct mediastinal invasion, and multi-station N2 disease—have to date been excluded from neoadjuvant trials. Thus, it is more difficult to have a complete perspective on the outcome and on the technical surgical aspects in this set of patients. In fact, if considering the possibility of extensive reconstructive surgery, even the patients considered to have unresectable disease could benefit from neoadjuvant therapy, with the possibility to then undergo extended curative resection. Patients with locally advanced lung cancer face the problem of R0 resection; in fact, bilobectomy or pneumonectomy may lead to a poor quality of life, and the alternative for preserving patients’ performance status after surgery could be the choice of an R1/R2 resection, with the limitation of leaving residual tumors and thus worsening the overall survival [37]. Nonetheless, extended resections could be considered as a valid alternative.

To date, an objective assessment of the effect of neoadjuvant chemo-immunotherapy considering the technical difficulty of lung resection associated with the therapy has not yet been conducted. Some surrogates used to define surgical complexity include time of surgery, the amount of intra-operative blood loss, rate of conversion from minimally invasive to open surgery, and post-operative complications.

Sleeve lobectomy is now widely accepted as a safe procedure to achieve both complete resection of tumors invading the central structures and the parenchymal sparing technique. According to the literature, this specific kind of surgery offers better short-term recovery and long-term survival outcomes than pneumonectomy [17]. Nevertheless, the feasibility and the safety of sleeve lobectomy after neoadjuvant chemo-immunotherapy is not widely reported in the literature. Some studies reported preliminary experiences of sleeve lobectomies performed in patients who underwent neoadjuvant chemo-immunotherapy, and no increased surgical difficulties were reported when compared to a standard population undergoing upfront surgery without neoadjuvant therapy [38].

Citing Minervini et al., “better conversion than complication”; in fact, regarding the specific surgical approach, the best approach should be identified considering tumor characteristics, surgeon confidence, and timing. In the era of minimally invasive surgery, reducing the amount of surgical access is not indicative of a reduction in complications. Therefore, surgeon personal security gives the safest result for patients, and it is extremely important in this difficult set of patients after neoadjuvant chemo-immunotherapy, potentially at risk for increased technical intra-operative difficulty. In fact, innovative surgery should aim not only to achieve a minimally invasive surgery, but above all, it should aim to limit organ disfunction, by preserving lung function as much as possible, thus improving the patient’s quality of life while prolonging survival [20,21,28,35,39,40,41,42,43,44]. Bulky tumors, infiltration of surrounding stricture, and/or mediastinum can be considered predictive factors for conversion, and open surgery is suggested for these kinds of lesions.

The present study presents some limitations: heterogeneity in study methodologies, a lack of standardized intra-operative assessment metrics, and a need for prospective trials focusing specifically on surgical feasibility.

Moreover, further studies analyzing the pathologic assessment of tumor response to neoadjuvant therapy would be integral to the multidisciplinary management of non-small-cell lung cancer (NSCLC), providing critical insights into tumor regression and its correlation with clinical outcomes. Major pathological response (MPR), defined as less than 10% residual viable tumor cells post-therapy, has emerged as a key prognostic marker associated with improved survival [20]. Similarly, pathological complete response (pCR), characterized by the absence of viable tumor cells, has been demonstrated to strongly predict favorable long-term survival [41]. An important aspect of post-treatment assessment is the evaluation of lymph node regression following neoadjuvant therapy. Studies have highlighted that lymph node response is a key determinant of prognosis, particularly in patients with clinical N2 disease. The evaluation of pathologic response in lymph nodes, including fibrosis and the extent of viable tumor cells, provides additional prognostic stratification and may inform post-surgical therapeutic decisions [28].

Imaging modalities such as RECIST 1.1 remain widely utilized for response evaluation; however, discrepancies between radiographic and histopathologic responses are well documented, especially in the setting of neoadjuvant immunotherapy. Residual radiologic abnormalities may not accurately reflect viable tumor burden due to therapy-induced fibrosis and immune-related changes [42]. This discrepancy underscores the necessity of histopathologic validation to guide therapeutic strategies and avoid premature clinical conclusions.

Recent clinical trials have reinforced the prognostic significance of histopathologic response in patients undergoing neoadjuvant immunotherapy-based treatments. The CheckMate 816 trial demonstrated a significant increase in pCR rates with neoadjuvant nivolumab plus chemotherapy compared to chemotherapy alone, highlighting the role of pathologic response assessment as a meaningful endpoint [3]. Moreover, perioperative strategies such as those explored in the AEGEAN trial continue to emphasize the evolving role of histopathologic response in refining treatment paradigms [43].

## 5. Conclusions

Neoadjuvant chemo-immunotherapy improves oncological outcomes but increases surgical complexity due to fibrosis and adhesions. Surgeons should anticipate longer operative times, higher conversion rates, and the necessity for systematic lymph node dissection. Standardized scoring systems for surgical difficulty should be incorporated into future trials to refine patient selection and optimize surgical outcomes.

## Figures and Tables

**Table 1:** Comparative table of scoring systems.

	Surgical Complexity	Grade
Lee et al. (2021) [7]	Lymphadenopathy	
	<1 cm	0
	1–2 cm	1
	2–3 cm	2
	>3 cm	3
	Grade of fibrosis	
	Mild fibrosis	1
	Moderate fibrosis increasing surgery complexity	2
	Severe fibrosis requiring conversion	3
	Severe fibrosis resulting in unresectability	4
	Site of lung cancer	
	Central	1
	Peripheral	2
	Perihilar/mediastinal adhesions	
	Mild fibrosis	1
	Moderate fibrosis increasing surgery complexity	2
	Severe fibrosis requiring conversion	3
	Severe fibrosis resulting in unresectability	4
Sepesi et al. (NEOSTAR study, 2019) [8]	Time of surgery	NA
	Intra-operative blood loss	NA
	Conversion rate	NA
Zhang et al. (2021) [9]	Intrathoracic adhesion	
	No adhesion	0
	Mild/moderate adhesions	1
	Severe adhesions	2
	Difficulty of nodes’ dissection	
	Normal	0
	Hard	1
	Extremely hard	2
	Grade of tumor invasion	
	No peripheral invasion	0
	Mild invasion of pulmonary artery (PA) and/or bronchus	1
	Severe invasion of the main PA and/or bronchus	2
	Difficulty of the whole procedure	
	Normal	0
	Hard	1
	Extremely hard	2

**Table 2:** Type of surgery, conversion rate, and post-surgical complications.

Trial	N Patients	Neoadjuvant	Type of Surgery	Surgical Approach	Conversion Rate	Complications
NEOSTAR Sepesi et al. [8]	37	Nivolumab	Lobectomy: 30/37 (81%) Sleeve lobectomy: 2/37 (5%) Bilobectomy: 1/37 (2%) Pneumonectomy: 2/37 (5%) Segmentectomy: 1/37 (2%) Wedge resection: 1/37 (2%)	Thoracotomy 27/37 (73%) VATS 7/37 (19%) RATS 3/37 (8%)	2/12 (17%)	21/37 (57%)
Zeng et al. [16]	220	PD-1/PD-L1 immune checkpoint inhibitors combined with platinum-based doublet chemotherapy	Lobectomy 163/220 (74%) Bilobectomy 31/220 (14%) Sleeve lobectomy 8/220 (3.6%) Pneumonectomy 18/220 (81.8%)	VATS 78/220 (35.4%) RATS 142/220 (64.5%)	VATS 62/220 (28.2%) RATS 17/220 (7.5%)	71/220 (32.3%)
CheckMate 816 Forde et al. [3]	179	Nivolumab + platinum doublet chemotherapy	Lobectomy 115/179 (78%) Pneumonectomy 25/179 (17%) Sleeve lobectomy 2/179 (1.3%) Other 24/179 (16%)	Thoracotomy 88/179 (59%) Minimally invasive 44/179 (29.5%)	17/179 (11.4%)	62/149 (41.6%)
Liang et al. [17]	10/20	PD-1/PD-L1 immune checkpoint inhibitors combined with platinum-based doublet chemotherapy	Sleeve lobectomy 100%	Thoracotomy 3/10 (30%) VATS 7/10 (70%)	3/10 (30%)	0/10 (0%)
Zhu et al. [18]	23	PD-1/PD-L1 immune checkpoint inhibitors combined with platinum-based doublet chemotherapy	Sleeve lobectomy 100%	Thoracotomy 15/23 (65%) VATS 8/23 (35%)	1/8 (12.5%)	3/23 (13%)
Chen et al. [19]	12	Nivolumab or Pembrolizumab + platinum doublet chemotherapy	Lobectomy 8/12 (66.7%) Bilobectomy 1/12 (8.3%) Sleeve lobectomy 3/12 (25%)	Thoracotomy 9/12 (75%) VATS 3/12 (25%)	0%	4/12 (33%)
Keynote 671 [20]	397	Pembrolizumab + chemotherapy	Pneumonectomy (9%)	NR	NR	
Neotorch [21]	202	Toripalimab + platinum doublet chemotherapy	Pneumonectomy (9%)	NR	NR	128/202 (63.4%)
SAKK 16/14 [22]	55	Durvalumab + cisplatin + docetaxel	Pneumonectomy: 5/55 (9%) Lobectomy: 43/55 (78%) Bilobectomy: 5/55 (13%)	NR	NR	17/55 (31%)

**Table 3:** Main reported post-operative complications.

Study	Pts	Type of Surgery	Complications
CheckMate 159 [35]	20	Thoracotomy: 14/20 (70%) Thoracoscopy: 3/20 (14%) RATS: 3/20 (14%) Conversion rate: 7/13 (54%)	Trasfusion 0% Pnemonia 1/20 (5%) Bronchopleural fistula 0% PAL 1/20 (5%) Resp failure 0%
NEOSTAR [8]	37	Thoracotomy: 27/37 (73%) VATS: 7/37 (19%) RATS: 3/37 (8%) Conversion: 2/12 (17%)	Trasfusion 0% Pnemonia 1/21 (5%) Bronchopleural fistula 1/21 (5%) PAL 5/21 (24%) Resp failure 0%
NADIM [30]	41	NR	Trasfusion 0% Pnemonia 0% Bronchopleural fistula 5/41 (12%) PAL 0% Resp failure NR
Zhu et al. [18]	23	Sleeve lobectomy 100%	Pulmonary Infection 1/23 (4.3%) Acute Coronary Syndrome 1/23 (4.3%) Bronchopleural Fistula 1/23 (4.3%)
Chen et al. [19]	12	Lobectomy 8/12 (66.7%) Bilobectomy 1/12 (8.3%) Sleeve lobectomy 3/12 (25%)	Trasfusion 0% Pnemonia 0% Chylothorax 1/12 (8.3%) Bleeding 1/12 (8.3%) PAL 1/12 (8.3%)
